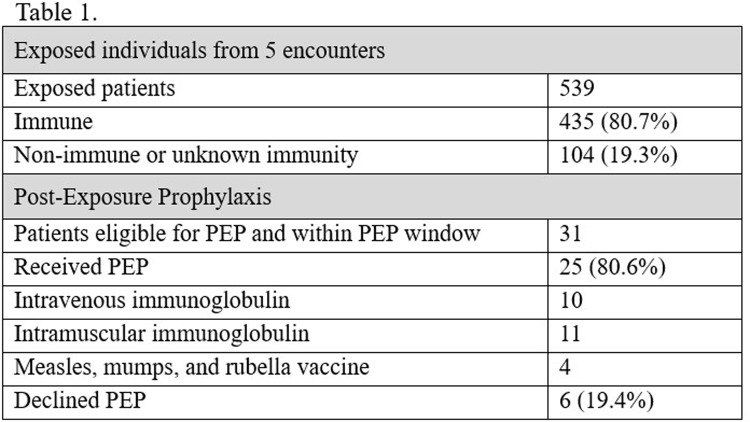# 35 Etiologic Agents of Non-COVID Community-Acquired Pneumonia Requiring Hospitalization – Analyzing Changes Over Time

**DOI:** 10.1017/ash.2026.10710

**Published:** 2026-06-23

**Authors:** Jean Barth, Leah Siple, Alyssa Olson, Jenna Rasmusson, Rebecca Faller, Michelle Meyer, Becky Baxter, Emily Loberg, Debra Apenhorst, Leah Higbe, Sarah Bellows Mahler, Sheryl Maurer, Heidi Whitehurst, Felice Paden, Sheila Higbe, Elena Beam, John OHoro, Emily Levy, W. Charles Huskins, Priya Sampathkumar

**Affiliations:** 1 Mayo Clinic; 2 Mayo Clinic Rochester; 3 Mayo; 4 Mayo Clinic College of Medicine; 5 Mayo Clinic, Rochester, MN

## Abstract

**Background:** Measles is a highly transmissible virus. A single case can result in significant exposures within a healthcare facility and prompt action is needed to prevent secondary cases. In October 2025, Infection Prevention and Control (IPAC) was notified of a patient being admitted to our facility with recent international travel and symptoms consistent with measles. The patient was unvaccinated and had five healthcare facility visits prior to measles being suspected and subsequently confirmed. Objective: To describe our facility’s effective identification, prioritization, and prophylaxis of individuals exposed to measles. **Methods:** IPAC leveraged the electronic medical record to identify potentially exposed patients. Patients were quickly assessed for age and immune status, then prioritized and assigned to primary care teams. These teams promptly contacted patients and coordinated PEP administration. **Results:** Across five encounters, the index patient exposed 539 other patients. Due to delayed notification, our teams had less than four days to administer PEP for those exposed and still within PEP window. PEP administration began within hours of the index case’s positive test and patients received PEP within three days (Table 1). No secondary measles cases occurred within our facility. **Conclusion:** Timely and coordinated multidisciplinary effort enabled us to rapidly respond to our first measles case and control secondary transmission. Although the index case resulted in a large exposure, patients that were exposed and eligible for PEP were quickly prioritized to prevent secondary transmission.

**Figure:**